# Economic evaluation of a national vitamin D supplementation program among Iranian adolescents for the prevention of adulthood type 2 diabetes mellitus

**DOI:** 10.1186/s12906-021-03474-0

**Published:** 2022-01-03

**Authors:** Narges Zandieh, Mohsen Rezaei Hemami, Ali Darvishi, Seyed Mohammad Hasheminejad, Zahra Abdollahi, Maryam Zarei, Ramin Heshmat

**Affiliations:** 1 Faculty of pharmacy, Tehran Medical sciences, Islamic Azad University, Tehran, Iran; 2 University of Aberdeen, Aberdeen Centre for Health Data Sciences, Aberdeen, UK; 3 Department of Management and Health Economics, School of Public Health, Tehran University of Medical Sciences (TUMS), Tehran, Iran; 4 Department of Management, Medical Science Branch, Islamic Azad University, Tehran, Iran; 5 Office of Community Nutrition, Deputy of Health, Iran Ministry of Health and Medical Education, Tehran, Iran; 6 Chronic Diseases Research Center, Endocrinology and Metabolism Population Sciences Institute, Tehran University of Medical Sciences, Tehran, 1941933111 Iran

**Keywords:** Vitamin D supplementation, Cost-effectiveness, National program, Diabetes management

## Abstract

**Background:**

This study aimed to evaluate the cost-effectiveness of vitamin D supplementation in preventing type 2 diabetes mellitus (T2DM) among Iranian adolescents.

**Methods:**

This analytical observational study was conducted, using the decision tree model constructed in TreeAge Pro to assess the cost per quality-adjusted life-year (QALY) of monthly intake vitamin D supplements to prevent T2DM compared to no intervention from the viewpoint of Iran’s Ministry of Health and through an one-year horizon. In the national program of vitamin D supplementation, 1,185,211 Iranian high-school students received 50,000 IU vitamin D supplements monthly for nine months. The costs-related data were modified to 2018. The average cost and effectiveness were compared based on the Incremental Cost-Effectiveness Ratio (ICER).

**Results:**

Our analytical analysis estimated the 4071.25 (USD / QALY) cost per AQALY gained of the monthly intake of 50,000 IU vitamin D for nine months among adolescents over a one-year horizon. Based on the ICER threshold of 1032–2666, vitamin D supplementation was cost-effective for adolescents to prevent adulthood T2DM. It means that vitamin D supplementation costs were substantially less than the costs of T2DM treatments than the no intervention.

**Conclusions:**

Based on the findings, the national vitamin D supplementation program for Iranian adolescents could be a cost-effective strategy to reduce the risk of diabetes in adulthood. From an economic perspective, vitamin D supplementation, especially in adolescents with vitamin D deficiency, would be administrated.

## Introduction

Type 2 diabetes mellitus (T2DM) is a major chronic disease that has been considered a serious public health concern worldwide. According to statistics, over 500 million people were diagnosed with T2DM up to 2019 around the world. The worldwide prevalence of diabetes is increasing, especially in Iran, which was reported a 35% increment in 2011 compared to 2005 [1]. T2DM complications negatively affect the patient’s health and are related to the worsening health-related quality of life, which the estimates show that more than four million diabetic patients died in 2017 alone [2]. The health-care costs of a diabetic patient are 2.5 times higher than a healthy person in Iran [3].

Vitamin D deficiency is one of the most common causes of chronic diseases such as T2DM these days. It was suggested that vitamin D deficiency in children could increase the risk of diabetes in adulthood [4]. Vitamin D deficiency may play a key role in reducing insulin secretion from pancreatic β-cells, increasing the inflammatory markers and insulin resistance [5]. There is a substantial discrepancy in recently-conducted studies. Some studies revealed that vitamin D supplementation negatively affects glucose hemostasis [6–11], while some clinical trials did not significantly affect [12–14]. Based on the reports of the third National Health and Nutrition Examination Survey (NHANES III) among 6228 individuals, higher levels of serum vitamin D are associated with a lower risk of T2DM development [15]. Besides the increasing prevalence of T2DM in Iran, a national study on the serum micronutrient status reported that 76% of Iranian adolescents suffer from vitamin D deficiency. The findings of this study revealed that the prevalence of vitamin D deficiency in children and adolescents varied from 46.8% in summer to 100% in winter in regions with different climates [16]. Considering the low level of serum vitamin D in Iranian adolescents, a vitamin D supplementation program was implemented in 2014 to increase their serum vitamin D level. In this program, the students receive 50,000 IU vitamin D/monthly for nine months of the year. Performing a nationwide supplementation program imposes a heavy burden on the health-care system and economy, especially in a developing country. Therefore, the cost-effectiveness of such programs should be determined. The results of cost-effectiveness analysis (CEA) can help health policy makers allocate resources better and implement supplementary programs [17, 18]. The CEA analysis compares the costs and benefits of two strategies of supplementation and no intervention with each other, and Incremental Cost-Effectiveness Ratio (ICER) is usually used to show the results of the analysis [19]. The Quality-Adjusted Life Year (QALY) is one of the most reliable variable to show the results of cost-effectiveness [20]. To the best of our knowledge, no cost-effectiveness analysis has been performed to determine the cost and benefits of vitamin D supplementation in adolescents to reduce the risk of T2DM in adulthood, so we aimed to examine the cost-effectiveness of vitamin D supplementation in the prevalence of diabetes in adolescents with improved vitamin D levels. The current analysis was conducted from the Ministry of Health and Medical Education (MOHME) perspective because the MOHME is responsible for paying for the medical approaches like the national vitamin D supplementation program based on their well-documented evidence. The study’s objective was to capture the cost-effectiveness of a nationwide vitamin D supplementation among Iranian adolescents to prevent T2DM in the older ages.

## Materials and methods

### National program’s characteristics

#### Target population and setting

In this study, we randomly selected the data of 1,519,762 students (733,657 females and 786,105 males) in the first or second grade of high school in 47 climatically different cities of Iran. The crude data about the total number of students and schools covered by the vitamin D supplementation program in 2018 was provided by the Nutrition Improvement Office of the Ministry of Health. Considering the higher prevalence of vitamin D deficiency among girls [21], 87.5% of girls and 69.5% of boys were covered by the vitamin D supplementation program. Overall, 78.5% of the total population, 1,185,211 students, received vitamin D supplements.

### Supplementation strategy

In the national vitamin D supplementation program, one pearl of 50,000 IU vitamin D per month was considered for each student for nine months. Each student was asked to use one vitamin D pearl every month during autumn, winter, and spring. Considering the possibility of vitamin D toxicity, this program was implemented over nine months of the year, except for summer.

### Comparators and time horizon

In this analytical observational study, we used a cost-effectiveness analytical approach method. Two strategies were examined. The first strategy consists of no complimentary assistance or intervention. The second strategy includes vitamin D supplementation with 50,000 IU Vitamin D monthly for nine months by the Ministry of Health as part of the supplement program. We applied these two vitamin D supplementation strategies versus no intervention to show that the national one-year vitamin D program for Iranian adolescents is cost-effective to prevent adulthood diabetes. A one-year horizon was considered to determine the monthly intake of 50,000 IU vitamin D supplementation for nine months on costs and quality of life.

### Choice of health outcomes and *effectiveness measurement*

To measure the health outcomes of the vitamin D supplementation program, we considered the values of QALYs for healthy individuals and patients with T2DM. To calculate the QALYs values, we calculated the average of the utility value estimates in patients with T2DM [22, 23] or healthy ones [17, 24] as reported in previous studies. Based on a study conducted by Najmeh Moradi and her colleagues in 2019, which was the only study to calculate the QALYs values of diabetics in Iran, the results of the EQ-5D-3L questionnaire showed that the rate of Utility in diabetic people is about 0.51 ± 0.24. Moreover, to estimate the utility values for healthy ones, two studies conducted by Goodarzi and his colleagues in Iran during 2016 and 2019, using VAS and TTO methods, were used. This study was the first and most comprehensive study conducted in Iran and is the reference one for most utility studies in Iran. In these studies, the QALYs values was calculated to be 0.76.

In our study, the students were categorized as vitamin D deficient or vitamin D sufficient if the serum levels of their vitamin D were less than 30 ng/mL or more than 30 ng/mL, respectively [25]. The possible association between different serum levels of vitamin D and the prevalence of T2DM was determined based on a previous study [26].

The present analytical study was conducted in a time frame in the age group of Iranian adolescents and young people who have been covered by the vitamin D supplementary national program.

### Costs estimation

Based on the Table 1, the total project associated costs included both direct medical and non-medical costs. The direct medical costs included the acquisition of vitamin D pearls, while the direct non-medical costs included vitamin D supplementation administration, such as training the executors and controlling and supervising the program process. To account to this, we collected the required data from the Nutrition Improvement Office of Iran’s Ministry of Health and modified them to 2018. Based on the reports of the Central Bank of the Islamic Republic of Iran, the inflation rate in 2018 was equal to 9.6, so we finally converted the total estimated project costs to US dollars (USD) by using the mentioned conversion rate [28].

**Table 1:** Data related to The Probabilities, Costs and Expenditures^a^

Descriptions	Values
**Probability**	
The probability of vitamin D deficiency before starting the supplementation program [16]	76%
The probability of vitamin D sufficiency before starting the supplementation program [16]	24%
The probability of vitamin D deficiency after starting the supplementation program [27]	17.2%
The probability of vitamin D sufficiency after starting the supplementation program [27]	87.2%
The prevalence of diabetes incidence in people with vitamin D deficiency [26].	12.8%
The prevalence of diabetes incidence in people with adequate vitamin D levels [26].	7%
**Cost** ^b^	
The costs of the 9 pearls of vitamin D for each student	0.236–0.472USD
The costs of the training staff to perform or supervise the project	0.25 USD
The total costs of a one-year national program of vitamin D supplementation	0.499–0.72 USD
The 2018-adjusted costs of diabetes management for each patients during one year	709 USD

^a^ The expenditure data in 2018 was calculated at a dollar exchange rate declared by Iran’s Central Bank

^b^According to Iran’s Central Bank reports, the inflation rates in 2017 was 9.6

According to the National Diabetes Statistics Report by the Iranian Ministry of Health in 2016, the cost of diabetes in 2014 was equal to 18,317,140 Iranian Rials per patient [29]. By considering Iran’s inflation rate in 2018, the 2018-adjusted costs of T2DM management would be about 19,111,120 Iranian Rials or 709 USD for each patient during one year.

### Economic analyses and results reporting

**Fig. 1:**
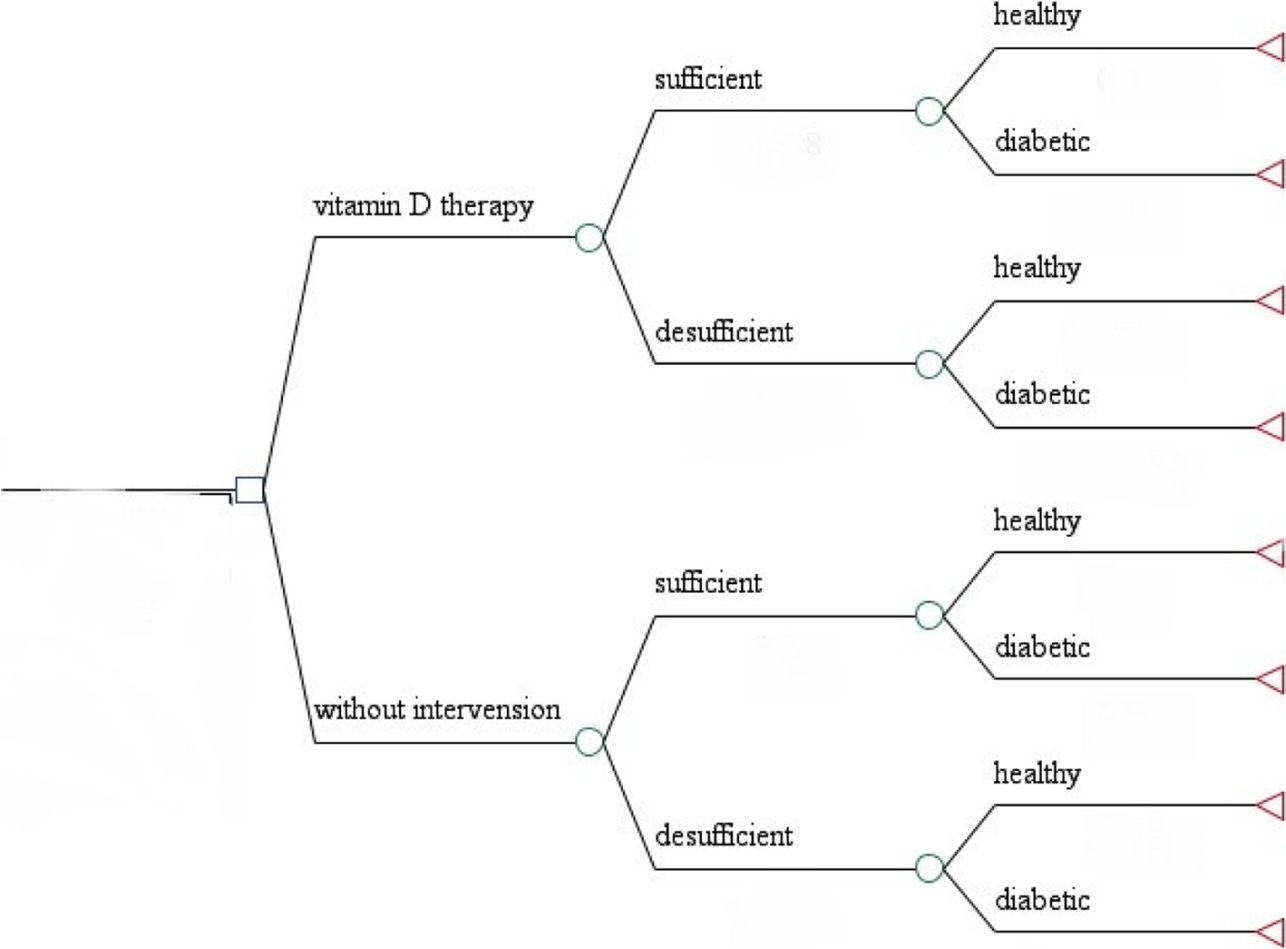
The decision tree model diagram (vitamin D intervention vs. no intervention)

In the current study, the vitamin D supplementation’s cost-effectiveness was compared to no intervention through a decision tree model (as shown in Fig. 1). The model was constructed in Microsoft Excel to evaluate. Excel software was used for initial data preparation, initial cost calculations, collection and extraction of parameter values. Over the other kinds of analytical models, the decision tree model is more understandable and easier to follow model with a logical approach, which provides different branches for different serum levels of vitamin D and presents each subject’s information [30]. We programmed the decision tree model and performed the sensitivity analysis through TreeAge Pro Inc. 2011 software 4.0.

The final results of costs and utilities are expressed in terms of ICER. The ICER can be measured as the differences between the total costs of vitamin D supplementation and no intervention divided by the difference between the related values of QALYs for them, as the following equation [31]. The ICER reveals the cost of vitamin D supplementation (compared to no supplementation) per each QALY gained.

$$=\frac{\mathrm{Costs}\ \mathrm{of}\ \mathrm{intervention}-\mathrm{Costs}\ \mathrm{of}\ \mathrm{no}\ \mathrm{intervention}\ }{\mathrm{QALYs}\ \mathrm{of}\ \mathrm{intervention}-\mathrm{QALYs}\ \mathrm{of}\ \mathrm{no}\ \mathrm{intervention}}$$

Furthermore, to discuss the cost-effectiveness of vitamin D supplementation in preventing T2DM more effectively, we compared the ICER value with a cost-effectiveness threshold. This threshold is the maximum level of the willingness to pay (WTP) per QALY gained. Based on the related study about the estimated WTP for QALY in Iran [32], we considered the range of 1032–2666 as the threshold of ICER.

### Assumption

According to recent studies, a monthly intake of one pearl of vitamin D (50,000 IU) is effective increasing the vitamin D’s serum level [5, 33] and maintaining the glucose hemostasis [8, 10, 12]. Based on the large body of evidence, childhood vitamin D deficiency develops some chronic illnesses such as T2DM in adulthood [4]. It was assumed that the vitamin D supplementation program among adolescents is a cost-effective strategy to reduce T2DM risk in adulthood.

### Sensitivity analysis

Since the pearls of vitamin D costs varied in 2018 and 2016, a sampling probabilistic sensitivity analysis was performed to assess the effects of the variations in the price changes of the vitamin D pearls, the cost of T2DM treatment, and the estimated values of utility based on the previous articles. To do this, the Monte-Carlo method and 1000 sampling were constructed to show the cost-effectiveness of vitamin D supplementation compared to no supplementation in T2DM prevention.

## Results

### Effectiveness

By considering the total sample size of 1000 individuals in this study, 500 participants had received the vitamin D supplementation and no intervention was considering for the rest. The analytical model showed that in the vitamin D supplemented participants, 39 people developed diabetes and 461 were healthy, while in the no-intervention group, 57 people had diabetes and 443 were healthy. According to the available evidence, the rate of desirability in diabetic patients was equal to 0.51 ± 0.24 QALYs and in normal people was 0.76 QALYs.

In 2020, Ganji et al. [26] estimated that 12.8% of individuals with serum vitamin D deficiency might develop diabetes, while in vitamin D sufficient individuals, this prevalence would reduce by 7%. It can be concluded that the prevalence of diabetes in individuals with deficient levels of vitamin D was about 1.8 times higher than that of normal subjects (Table 1).

Moreover, based on the previous studies showed in Table 1, 76% of Iranian adolescents were vitamin D deficient before the vitamin D supplementation program [16]. A monthly intake of 60,000 IU vitamin D for nine months can reduce the prevalence of vitamin D deficiency among adolescents up to 17.2% [27]. So it seems that vitamin D supplementation in adolescents might be effective in T2DM prevention in older ages.

### Estimated costs

As shown in Table 1, According to the MOHME reports in 2018, the direct medical costs to purchase nine vitamin D pearls ranged from 9908 to 19,816 Iranian Rials (equivalent to 0.236–0.472 USD). The direct non-medical cost to perform or supervise the program was about 11,049 Iranian Rials (0.25 USD). So, the total costs of the national vitamin D supplementation program for each student have been estimated to 20,957 to 30,865 Iranian Rials (0.499–0.72 USD) (Table 1).

### Cost-effectiveness analysis

The estimated cost per QALY gained of the vitamin D supplementation program was equal to 4071.25 (USD / QALY), and it is consistent with the expected QALYs threshold of 1032 to 2666, which means that the total costs of vitamin D supplementation were less than the costs of T2DM treatment of the no intervention (Table 2). It seems that vitamin D supplementation in adolescents is a dominant strategy through the cost-saving and QALYs increment.

**Table 2:** Results of Base Case Cost-effectiveness Analysis

Strategy	Cost($)	Incr Cost($)^a^	Eff^b^	Incr Eff ^c^	ICER (USD / QALY)
No Intervention	80.9968	23.60384	0.74061	0.0058	4071.25
Vitamin D Supplementation	57.39296	0	0.7464	0	

^a^Incremental costs

^b^ Effectiveness

^c^ Incremental Effectiveness

Incr Cost and Incr Eff were about 23.60384 and 0.0058, respectively

### Sensitivity analysis

**Fig. 2:**
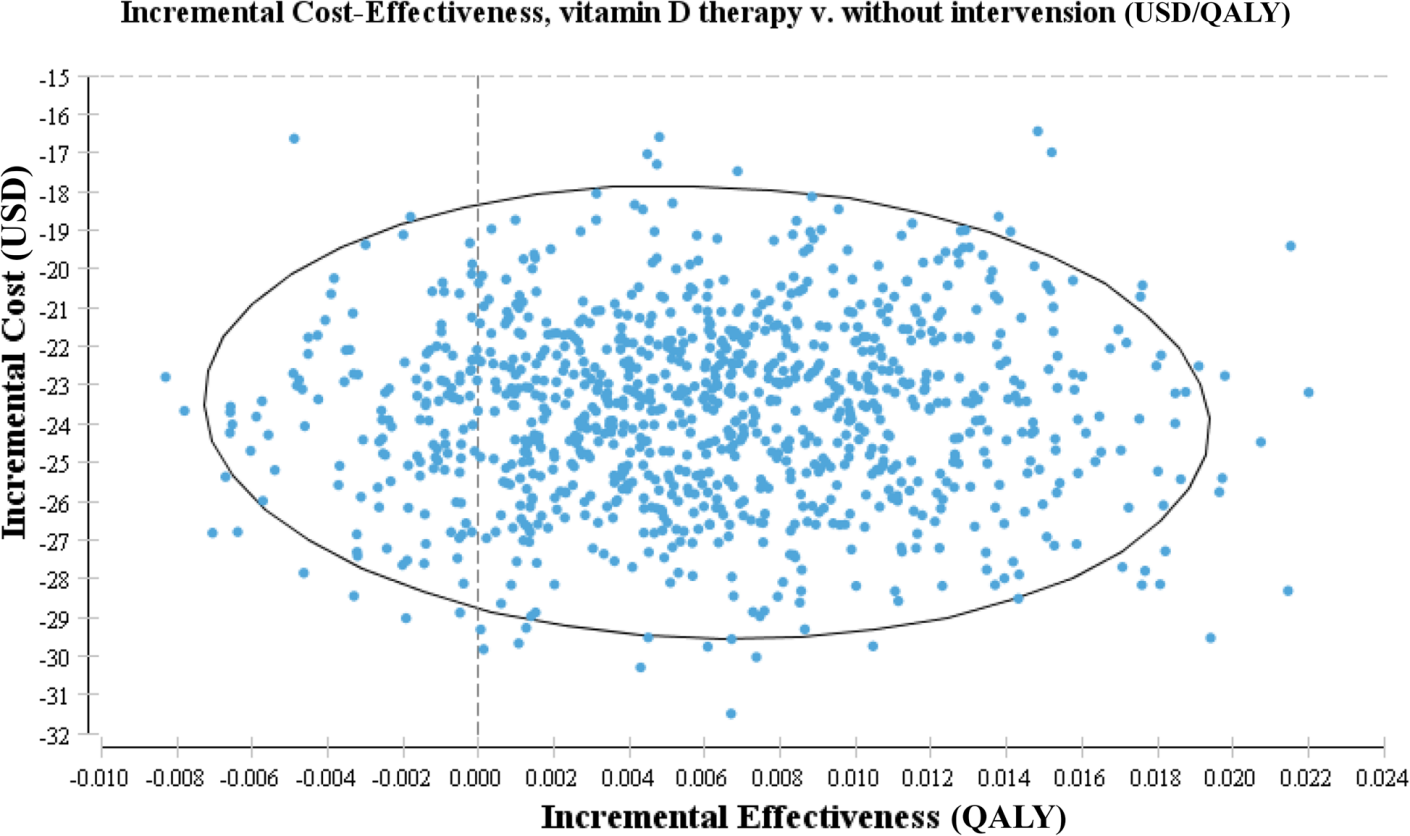
The incremental cost-effectiveness scatter plot (all samples are in the acceptable range)

**Fig. 3:**
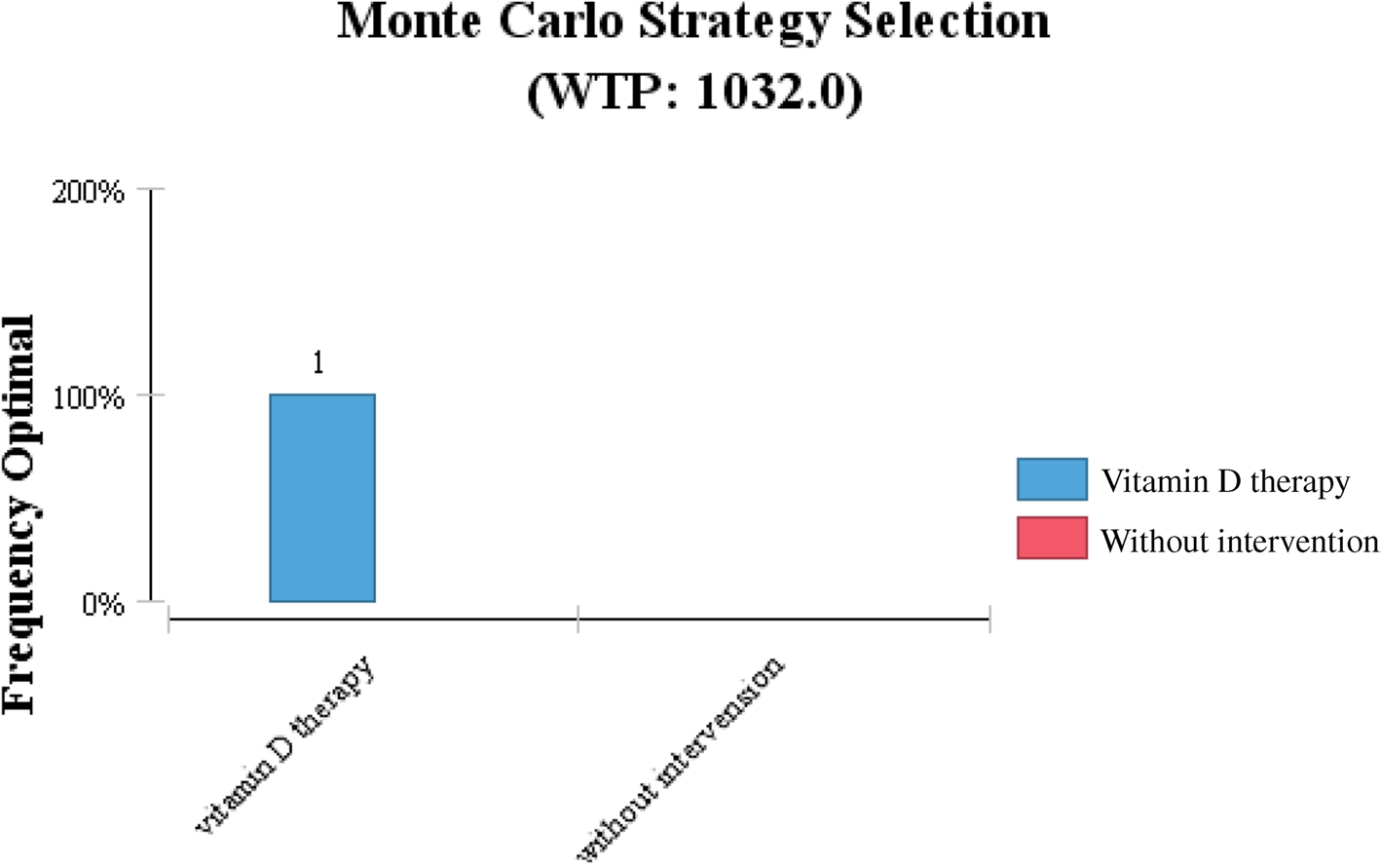
The Monte Carlo Strategy Selection (100% vitamin D therapy is dominant)

As shown in Table 3, performing the sensitivity analysis for model variables by the Monte-Carlo method showed that the ICER of vitamin D supplementation compared to no intervention is not sensitive to the parameters variation (i.e., costs of vitamin D pearls) and these changes could not affect the results. Therefore, vitamin D supplementation is the predominant and cost-effective strategy to prevent T2DM in adulthood with a 100% probability (as shown in Figs. 2 and 3). The study showed that modifying vitamin D levels both reduces the overall cost of diabetes management and increases utility, so the vitamin D modification strategy is the dominant strategy in diabetes prevention. In the probabilistic sensitivity analysis (Monte Carlo stimulation), the ICER value was set in in the same area after one thousand times of stimulation, so it was stated that it is 100%.

**Table 3:** Main Input Parameters of CEA Model

Statistic variable	Base case	SD/(CI)	Distribution	Source
*Costs($)*				
Cost of vitamin D therapy	0.61 USD	±0.12 USD	Gamma	*
Cost of diabetes	710 USD	±70 USD	GAMMA	[29]
*Utilities*				
Non-diabetes individual	0.76		Beta	[17, 24]
Individual with diabetes disease	0.59	±0.15	Beta	[22, 23]

^*^ the Nutrition Improvement Office of the Ministry of Health

## Discussion

The findings of this study propose that vitamin D supplementation for Iranian adolescents is highly cost-effective to reduce diabetes risk. The recent clinical trial justifies the effectiveness of vitamin D in T2DM prevention, which showed that monthly intake of 50,000 IU vitamin D in individuals with vitamin D deficiency decreases the progression rate toward overt diabetes [34]. We believed that the results of the current study could help healthcare policymakers better allocate financial resources and planning national diabetes prevention programs.

Based on our knowledge, the current economic study is the first one that assessed the cost-effectiveness of vitamin D supplementation in adolescents in diabetes prevention. The previous cost-effectiveness studies about vitamin D supplementation have mainly focused on osteoporosis [35, 36] and fractures [37–41].

Due to several reasons, including the skin melatonin content, inadequate dietary intake of vitamin D, increased indoor activities, limited exposure to sunlight, and air pollution, the vitamin D deficiency incidents are relatively high in the Iranian population [42]. In Iran, a national study on the serum micronutrient status reported that 76% of Iranian adolescents suffer from vitamin D deficiency (VDD). The findings of this study revealed that the prevalence of VDD in children and adolescents varied from 46.8% in summer to 100% in winter in regions with different climates [16]. Based on the National Nutrition and Health Survey in the United States reports in 2001–2004, adolescents with obesity with serum levels of vitamin D lower than 15 ng/ml are 3,8 times higher than those with vitamin D serum levels higher than 26 ng/ml [43]. In concordance with these reports, previous studies also showed that in children with lower levels of serum vitamin D, the cells are significantly resistant to insulin function [44], and in children and adolescents with the new onset of type 1 diabetes, the serum levels of vitamin D were significantly lower than the healthy ones at the time of diagnosis [45, 46]. A case-control study was conducted among 120 Egyptian children and adolescents and revealed a significant and positive correlation between vitamin D status and body mass index (BMI) [47]. In 2020, Ganji et al. showed that the prevalence of diabetes in Iranian individuals with vitamin D deficiency was 1.8 times higher than those with the normal vitamin D status (12.8% versus 7%) [26]. So, it seems that supplementation with vitamin D might be an effective strategy in T2DM prevention through the serum levels of vitamin D correction [34], and evaluation of their cost-effectiveness is essential. Different doses of vitamin D supplementation effectively increase the serum levels of vitamin D, daily intake of 100 IU [48] or weekly intake of 50,000 IU [34] vitamin D can increase the vitamin D serum levels by about 1 or 70 ng/ml, respectively. A monthly intake of 50,000 IU vitamin D is clinically safe, and in individuals without a laboratory-confirmed vitamin D deficiency, at least three months of supplementation is required to reach a desirable level [49].

Our finding also represents that the national program of monthly intake of 50,000 IU vitamin D for nine months in adolescents is predominant and cost-effective to prevent T2DM, which ICER is 4071 (USD / QALY) and crosses the WTP threshold of 1032 per QALY.

On the other hand, our sensitivity analysis demonstrates that variation in costs of vitamin D pearls and QALY values of diabetic or healthy individuals could not substantially affect the cost-effectiveness of the vitamin D supplementation national program. The present study has some potential limitations that should be noticed. First, we had no access to the data about students who dropped out of school, and our study’s analysis was restricted to those who are students. Second, based on our literature review, the serum level of vitamin D was not defined following this national supplementation program in any previous study; therefore, we used similar research data. Third, the exact adherence rate of participants in the vitamin D supplementation national program was not determined, which can be another potential parameter and impact the cost-effectiveness of the supplementation program. More researches and cost-effectiveness studies in the general population are needed to confirm our findings.

## Conclusion

This study suggested that the vitamin D supplementation national program among adolescents could be clinically a cost-effective strategy for diabetes prevention in adulthood. Our results will potentially help the healthcare policymakers reduce the healthcare system costs by implementing the vitamin D supplementation national program among Iranian adolescents and better financial resources allocation.

## Data Availability

The public access to the database used in this article is closed. Corresponding author received administrative permission to assess data. Still, if necessary, the data can be retrieved from the correspondent author or the Office of Community Nutrition Improvement, Ministry of Health of Iran or by correspondence with the relevant link. (https://nut.behdasht.gov.ir/%D8%A7%D8%B9%D8%B6%D8%A7%DB%8C-%D8%AF%D9%81%D8%AA%D8%B1-%D8%A8%D9%87%D8%A8%D9%88%D8%AF-%D8%AA%D8%BA%D8%B0%DB%8C%D9%87). Tt is important to remember, this is modelling study and used the result of secondary data for modelling. In this manuscript, we used secondary data from published in other journals to calculate the effectiveness and cost of diabetes management, cost of service calculated from labor office rules in Iran, with the coordination and assistance of the Office of Community Nutrition Improvement, Ministry of Health of Iran. The Iran’s Ministry of Health approves it medical assistance. Furthermore, to calculate the cost of diabetes managements, the national document of non-communicable diseases provided by Iran’s Ministry of Health was used.

## Abbreviations

CEA: Cost-effectiveness Analysis
ICER: Incremental Cost-Effectiveness Ratio
MOHME: Ministry of Health and Medical Education
NHANES: National Health and Nutrition Examination Survey
QALYs: Quality-adjusted Life Years
T2DM: Type 2 Diabetes Mellitus
WTP: Willingness-To-Pay
